# Polysaccharides from *Dolichos biflorus* Linn and *Trachyspermum ammi* Linn seeds: isolation, characterization and remarkable antimicrobial activity

**DOI:** 10.1186/s13065-017-0349-2

**Published:** 2017-11-21

**Authors:** Shibani Basu, Manojit Ghosh, Rupam Kumar Bhunia, Jhuma Ganguly, Bimal K. Banik

**Affiliations:** 1 Department of Chemistry, Indian Institute of Engineering Science and Technology, Shibpur, Howrah, West Bengal India; 2 Department of Metallurgy and Materials Engineering, Indian Institute of Engineering Science and Technology, Shibpur, Howrah, West Bengal India; 3 Advanced Laboratory for Plant Genetic Engineering (ALPGE) and Advanced Technology Development Center (ATDC), Indian Institute of Technology (IIT), Kharagpur, Kharagpur, India; 4 Research & Education Development, Community Health Systems of South Texas, 3135 South Sugar Road, Edinburg, TX 78539 USA

**Keywords:** Polysaccharides, Structural elucidation, Chemical characterization, Physiochemical identification, Antimicrobial activity

## Abstract

Polysaccharides are structurally complex and essential constituents of life, and therefore, studies directed to these kinds of molecules have received scientific attention. Despite an easy availability of *Dolichos biflorus* Linn and *Trachyspermum ammi* (Linn) seeds isolation, characterization and antimicrobial studies of polysaccharides derived from these two natural sources have not been investigated. Therefore, we report here isolation of polysaccharides, their purification and characterization from *Dolichos biflorus* Linn and *Trachyspermum ammi* (Linn) seeds. Gel permeation chromatography, GC–MS, SEM, XRD, EDX and FT-IR analyses show the presence of three pentose sugar such as d-ribose, d-arabinose, d-xylose and hexose sugar such as d-mannose, d-galactose and d-glucose. Unprecedented antimicrobial activity of these polysaccharides against Gram positive bacteria such as *Staphylococcus aureus* and *Bacillus subtilis* and Gram negative bacteria such as *Escherichia coli* and *Pseudomonas aeruginosa* are established.

## Introduction

There has been tremendous interest in the use of medicinal plants in developed as well as developing countries, because compounds obtained from medicinal plants have been shown to be effective sources of therapeutic agents, without undesirable side effects [1, 2]. Polysaccharides are very crucial since they have tremendous medicinal values. Current studies have proved that the structures of polysaccharides are closely related to their biological activities [3]. So elucidation of their structures is a fundamental objective for understanding structure–activity relationships and cause of these biological activities.

We report here an analysis of the structural composition of purified polysaccharides extracts and chemical nature of *Trachyspermum ammi* (Linn.) Sprague and *Dolichos biflorus* Linn in detail. Literature is found less secured on the elucidation of their structures of purified extracts of these species and studies of their significant biological activity.

*Trachyspermum ammi* (Linn) Sprague (Ajowan), belongs to the family of Apiaceae. Also known as bishop’s weed, it is an aromatic spice closely resembling thyme in flavor [4]. It is a native of Egypt and is distributed in the Mediterranean region and South-west Asia. It has long being used as the principal source of thymol *T. ammi* seeds and is employed as an antiseptic, aromatic, carminative and antioxidant source [5]. Its oil is used in the preparation of lotions and ointments in cosmetics industries and as a spice in many food preparations [6]. It has been reported to possess strong insecticidal activity, bronchodilatory effect on asthmatic airways and analgesic effect [7–9].

*Dolichos biflorus* Linn (Horsegram or Kulthi) is well known throughout India as a draught resistant crop. It also enriches the soil with nitrogen. So after harvesting the seeds, the soil can be ploughed with green manure [10]. *Dolichos biflorus* is a well known medicinal plant for its folk- medicinal properties. In herbal medicine, the seeds of it are mainly used as tonic, astringent, diuretic, and are also recommended in asthma, bronchitis, urinary discharges, hiccoughs, heart trouble and other diseases of the brain [11–13].

After isolation, both samples from the seeds of *T. ammi* and *D. biflorus* were purified, analyzed and compared by chemical and physico-chemical route. The percentage of active ingredients in the purified extract from the natural samples is dependent on geographical distribution as well as the environmental conditions such as temperature, rainfall, altitude, and hr of sun light exposure.

## Materials and methods

### Sample preparation

**Fig. 1:**
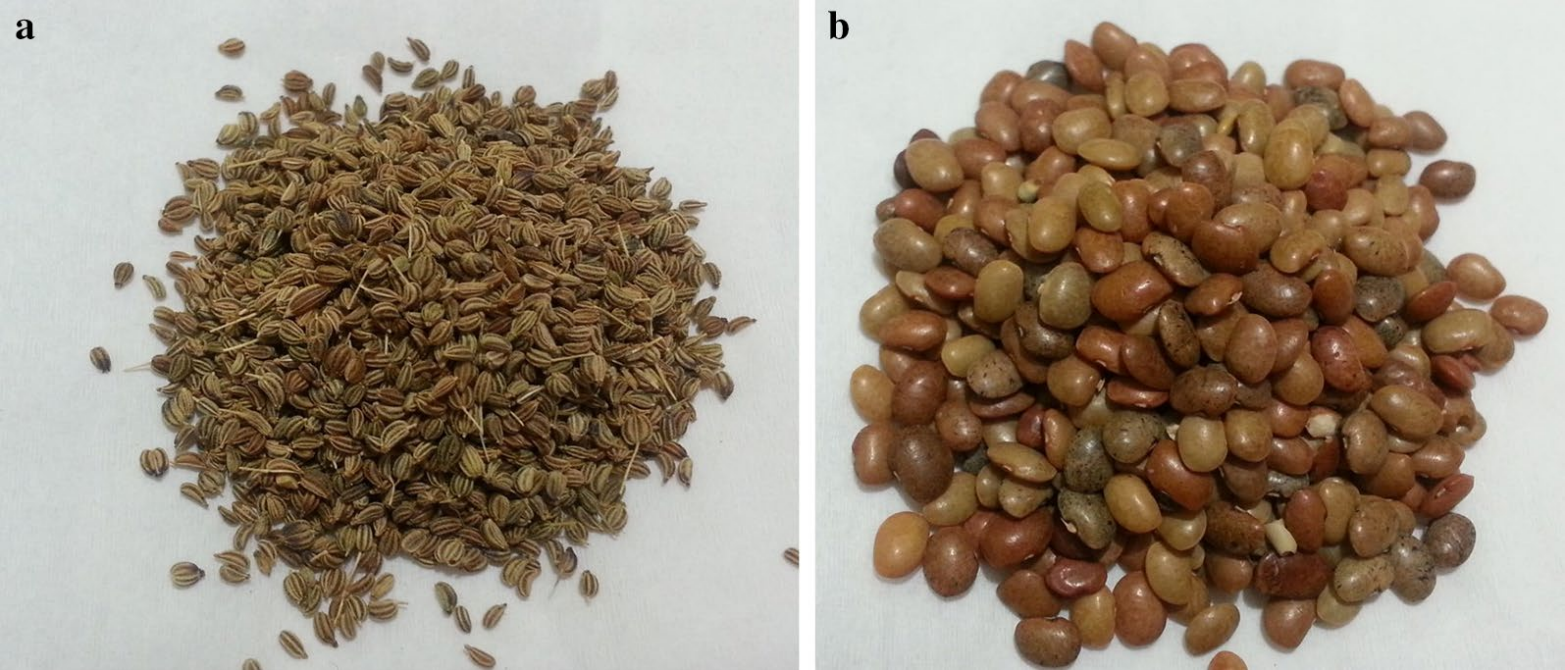
Seed of *Trachyspermum ammi* (Linn) Sprague (**a**) and seed and *Dolichos biflorus* Linn (**b**)

Seeds of *Dolichos biflorus* Linn and *Trachyspermum ammi* (Linn) Sprague were obtained from West Bengal, India and identified (Fig. 1, Table 1). Seed samples were prepared by extracting 10 gm of seeds of each sample separately in 50 mL of water and stirring for 48 h at room temperature (25 °C). The samples and named as TAE (*T. ammi* water extract) and DAE (*D. biflorus* water extract).

**Table 1:** Identification and authentication of samples

Sl. no.	Specimen/sample no.	Scientific name	Common name	Family name
1	J.G.1	*Trachyspermum ammi* (Linn) Sprague	Ajwain, ajoan, carom	Apiaceae; Alt. Umbelliferae
2	J.G.2	*Dolichos biflorus Linn*	Horse-gram, kulthi, hurali, Madras gram	Fabaceae; Alt. Leguminosae

### Isolation and purification of water soluble carbohydrate

The crude aqueous extracts were purified by gel permeation chromatography (GPC). The freeze-dried samples were dissolved in deionized water and applied to a Sephadex G-25 column (600 × 16 mm) that had previously been equilibrated with Mili-Q water at a flow rate of 0.3 mL min^−1^ and the column was then eluted with of Mili-Q water. Collected fractions were identified by phenol–sulphuric acid assay at 490 nm for the estimation of neutral sugar [14].

### Compositional analysis

The polysaccharides were hydrolyzed by TFA and derived by “acetic anhydride-pyridine” method. The derivatives were used to determine the monosaccharide compositions, because monosaccharides, in these conditions, were converted to volatile substances and therefore, they were easy to be detected by gas chromatography–mass spectrometry. Analytical GC and GC–MS were performed for quantitative and qualitative analysis of monosaccharides present in the purified fraction using alditol acetate derivatives with inositol as an internal standard [15]. Samples (200 µg) were hydrolyzed with 2N TFA (trifluoroacetic acid) at 120 °C for 2 h, NaBH_4_ (sodium borohydride) was used for reduction followed by 1 h incubation at 100 °C with acetic anhydride and pyridine (1:1). The hydrolyzed monosaccharides were then extracted with DCM (dichloromethane), analyzed on Agilent 6820 gas chromatograph equipped with HP-5 fused silica capillary column (30 m × 0.25 mm I.D). Mass spectra quantified on GC 7890A series equipped with MS 7000 GC/MS triple quad using DB-5 silica capillary column (30 m × 0.25 mm I.D). Nitrogen was used as the carrier gas (1.5 mL min^−1^). Detection was done by FID (300 °C) and identification by mass spectrometry held at 250 °C.

### FT-IR analysis

The major structural groups of purified extracts were detected using Fourier transform infrared spectroscopy (FT-IR). The FT-IR spectra were recorded in the region of 4000–400 cm^−1^ on a JASCO, FT/IR-460 PLUS using KBr pellet method. Background correction was made using a reference blank KBr pellet. The purified polysaccharides were ground with KBr powder by mortar and pestle. The mixture was then pressed into pellets for FTIR measurement in the frequency range of 4000–500 cm^−1^.

### SEM and EDX analysis

The samples were used for Scanning electron microscopy (SEM) analysis by fabricating a drop of suspension onto a clean electric stubs and allowing the solvent (i.e. water, methanol) to completely evaporate with platinum coating. The morphology of the purified extracts was observed on a ZEISS EVO 18 electron microscope with an accelerated voltage of 10–20 kV.

Elemental analysis of purified extracts was carried out using energy dispersive X-ray spectroscopy (EDX). The EDX of the purified extracts were measured by ZEISS EVO 18 electron microscope.

### XRD analysis

X-ray diffraction (XRD) analysis of drop-coated films of extracts was prepared for the study of phase and nature. A Bruker D-8 Advanced X-ray diffractometer with scanning range from 2θ = 20° to 80° and Ni-filtered Cu Kα radiation with wavelength 1.540598 Å used for this characterization.

### TGA−DTA analysis

Thermal analysis gives properties like enthalpy, thermal capacity, mass changes and the coefficient of heat expansion. TGA and differential scanning calorimetric (DTA) analysis were carried out by Perkin Elmer SII, Diamond TG/DTA machine. The samples were heated to 600 °C at a heating rate of 10 °C min^−1^, under flowing nitrogen of 10 mL min^−1^.

### Study of antimicrobial activity

The disc diffusion method was applied to evaluate the antimicrobial activity with four human pathogenic bacteria. Gram positive bacteria such as *Staphylococcus aureus, Bacillus subtilis,* Gram negative bacteria such as *Escherichia coli, Pseudomonas aeruginosa* were used in this study. 100 mL conical flask of nutrient broth was inoculated with the test organisms and incubated at 37 °C for overnight. By using a sterile pipette, 0.6 mL of the broth culture of each test organism was added to 60 mL of molten agar, which was cooled at 45 °C, mixed well and poured into a sterile Petri plate. Normal saline water was used as negative control and Tetracycline was used as positive control in comparison with test organisms. Then, the plates were left at room temperature for 2 h to allow diffusion of the test sample and incubated face upwards at 37 °C for overnight.

## Results and discussion

**Fig. 2:**
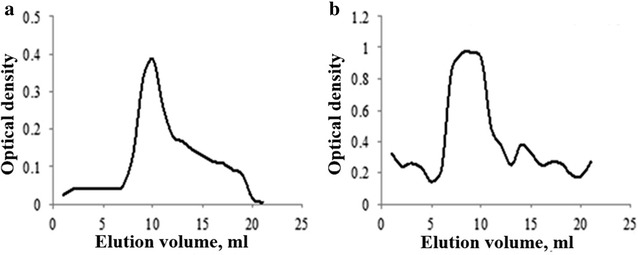
UV spectrophotometry analysis (the UV actives region at 490 nm) for the purity of water soluble polysaccharides of *T. ammi* (Linn) Sprague (**a**) and *D. biflorus* Linn. (**b**) purified by gel permeation chromatography

Isolation and purification by gel permeation chromatography (GPC) on a Sephadex G-25 column (600 × 16 mm) obtained UV actives region (at 490 nm) peaks for the phenol sulfuric acid assay (Fig. 2) from TAE and DAE (extracts were eluted with deionized water). As shown in Fig. 2, absorbance at 490 nm was observed, confirming that the isolated fractions were mainly polysaccharide rich.

**Fig. 3:**
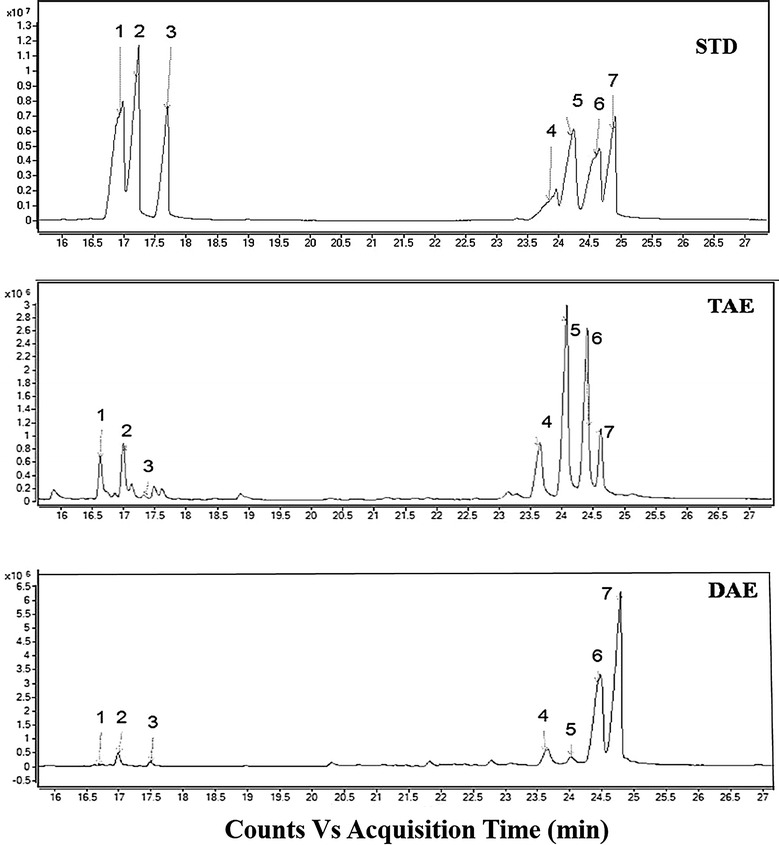
GC spectrum of standard sugar mixture (STD), TAE and DAE containing ribose (1), arabinose (2), xylose (3), inositol (4), mannose (5), glucose (6), galactose (7)

**Fig. 4:**
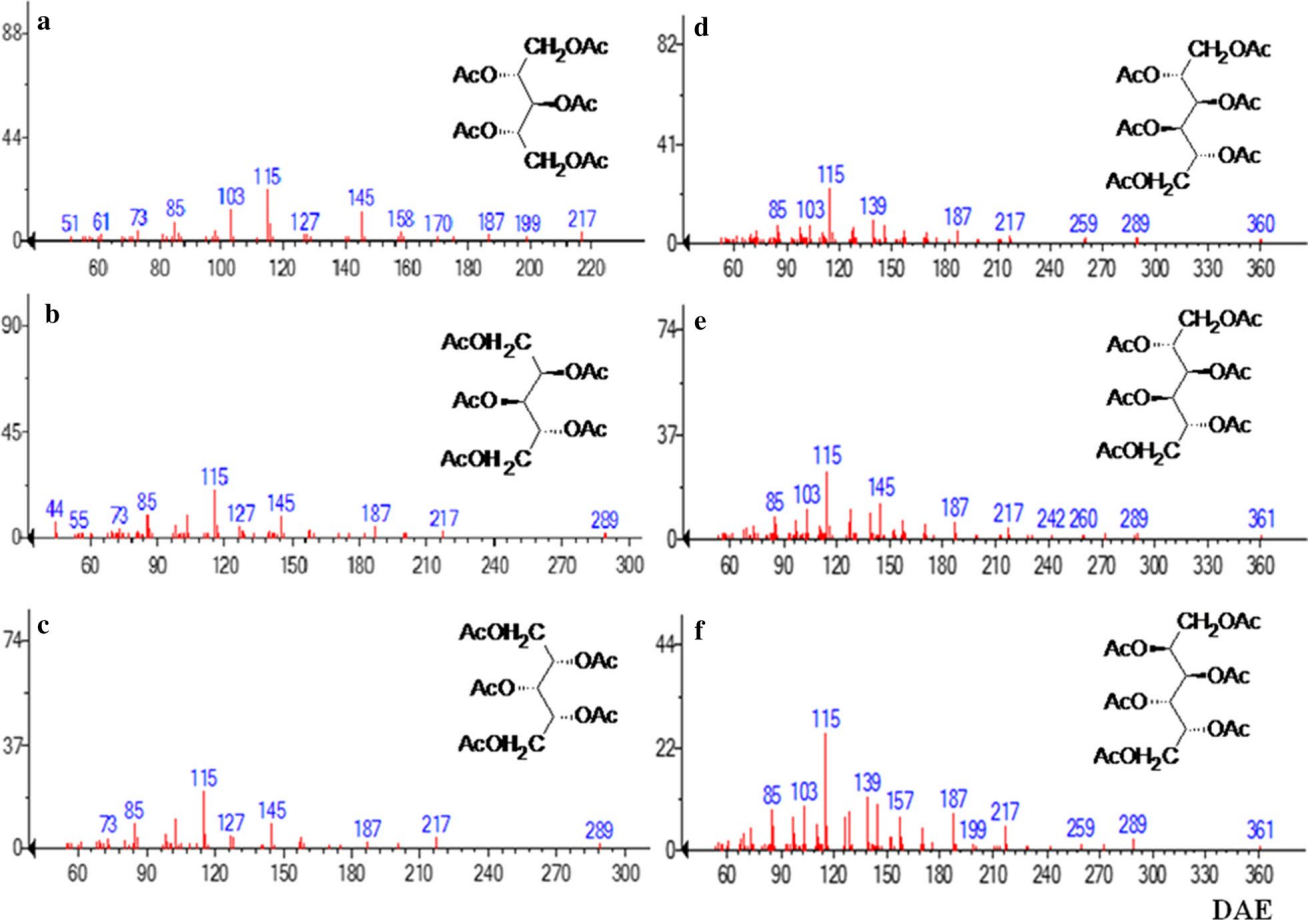
Mass fragmentation of TAE: 1,2,3,4,5-penta*-O*-acetyl ribitol (**a**); 1,2,3,4,5-penta *O-*acetyl arabinitol (**b**); 1,2,3,4,5-penta*-O-*acetyl *xylitol* (**c**); 1,2,3,4,5,6-hexa-*O*-acetyl mannitol (**d**); 1,2,3,4,5,6-hexa-*O*-acetyl glucitol (**e**); 1,2,3,4,5,6-hexa-*O*-acetyl galactitol (**f**)

GC–MS analysis determined six kinds of monosaccharides in TAE and four kinds of monosaccharide in DAE by comparing with standard sugar peak (Fig. 3). The GC and GC–MS analyses showed the presence of three pentose sugars such as d-ribose, d-arabinose, d-xylose and three hexose sugars such as d-mannose, d-galactose and d-glucose in TAE (Fig. 4). Importantly, the molecular ratios of the monosaccharide constituents in TAE were found to be present at 6:7:3:38.2:35.1:10.5 (Table 2).

**Table 2:** Molecular ratio of monosaccharide mixture from water soluble polysaccharides

Sample name	% of CHO	Compound presents # (mol%)					
		Arabinose	Ribose	Xylose	Mannose	Galactose	Glucose
TAE	70.05	6	7	3	38.2	35.1	10.5
DAE	70.09	2	–	1	2	37	58

**Fig. 5:**
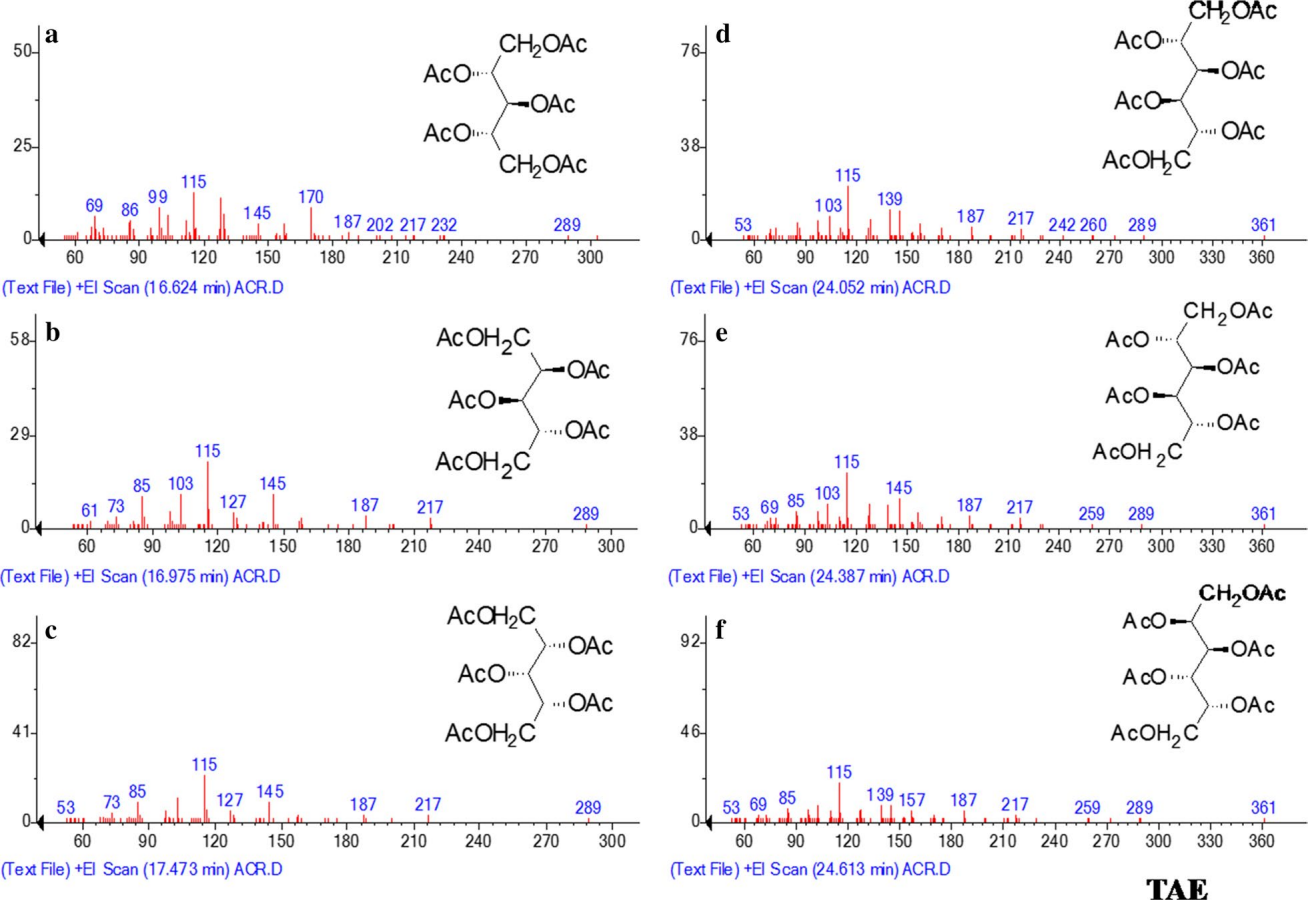
Mass fragmentation of DAE: 1,2,3,4,5-penta-*O*-acetyl ribitol (**a**); 1,2,3,4,5-penta *O*-acetyl arabinitol (**b**); 1,2,3,4,5-penta-*O*-acetyl xylitol (**c**); 1,2,3,4,5,6-hexa-*O*-acetyl mannitol (**d**); 1,2,3,4,5,6-hexa-*O*-acetyl glucitol (**e**); 1,2,3,4,5,6-hexa-*O*-acetyl galactitol (**f**)

On the other hand, by the GC–MS analysis in DAE (Fig. 5) confirmed the presence of d-arabinose, d-xylose as pentose sugar and d-mannose, d-galactose, d-glucose as hexose sugar with the molecular ratio of 2:1: 2:37:58 respectively (Table 3).

**Table 3:** Elemental study of purified extracts by EDX

Element	Sample	C	O	Mg	P	S	Cl	K	Ca	Sn
Weight%	TAE	45.67	46.76	0.54	0.30	0.27	0.91	3.67	1.35	0.53
	DAE	55.54	41.40	–	0.30	–	0.18	2.57	–	–
Atomic%	TAE	54.93	42.22	0.32	0.14	0.12	0.37	1.36	0.49	0.06
	DAE	63.41	35.49	–	0.13	–	0.07	0.90	–	–

**Fig. 6:**
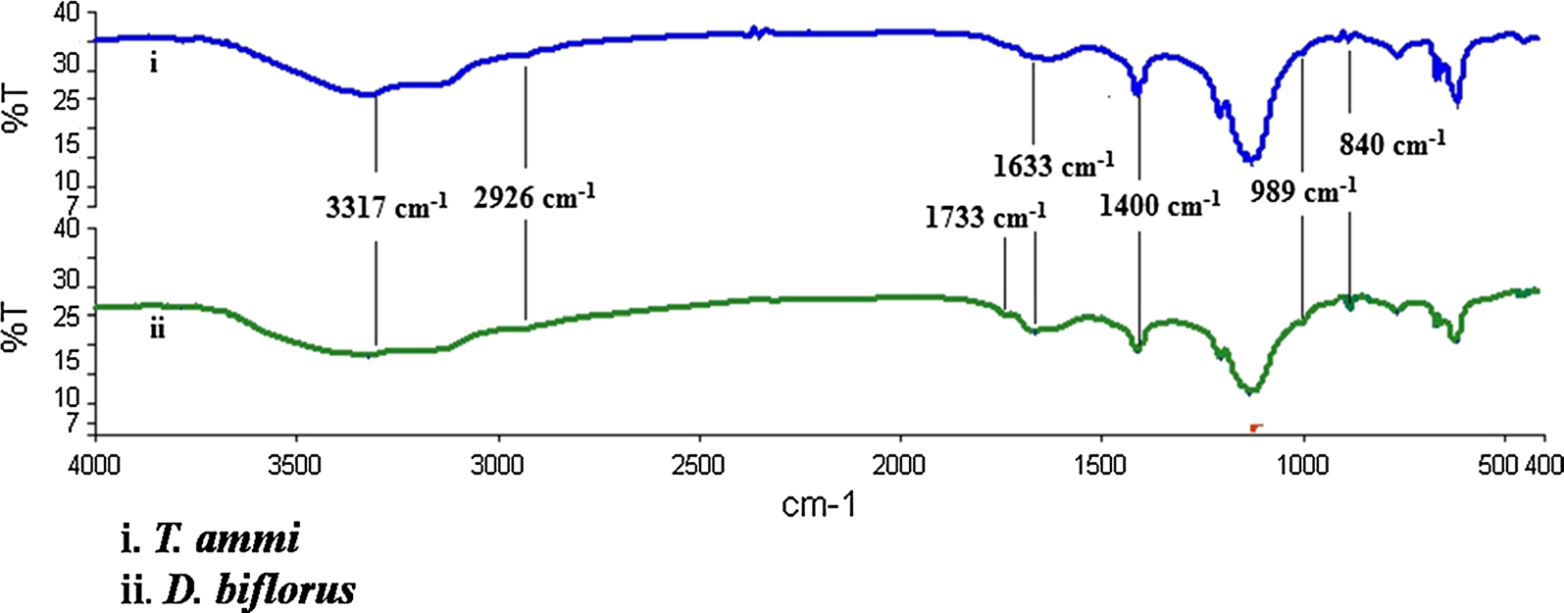
FTIR spectroscopy of purified sample

The functional group of purified sample was determined by FTIR spectroscopy using KBr pellet method (Fig. 6). The purified polysaccharides were ground with KBr powder and then pressed into pellets for FTIR measurement in the frequency range of 4000–500 cm^−1^. Dried TAE and DAE (water extract/methanol extract) showed a peak at 3317 cm^−1^ due to the stretching of the N–H bond of amino groups and indicative of bonded hydroxyl (–OH) groups alcohols and phenolic compounds. The absorption bands at 2926 cm^−1^ that appeared in spectrums were due to the stretching frequency of –CH_3_ groups. A peak at 1733 cm^−1^ in dried DAE indicated the presence of characteristic C=O stretching frequency of the carboxylic acid group. However, no such peak was present in TAE indicating the absence of –COOH group in it. It also shows absorption bands at 1633 and 1564 cm^−1^ for carbonyl stretching vibration (amide-I) or C=C groups/aromatic rings, N–H stretching vibration (amide-II), respectively for amide linkages of the proteins present in it, while medium broad band at 1400 cm^−1^ is the C–N stretching mode of aromatic amine group [16]. The absorption band at 989 cm^−1^ was due to the C–O–C vibrations of proteins/polysaccharides present in the extract [17]. The band at 840 cm^−1^ was assigned due to the in plane and out of plane bending for benzene ring. This indicated that extracts are surrounded by some proteins and metabolites such as alkaloids and terpenoids that have functional groups of amines, alcohols, ketones, aldehydes, and carboxylic acids.

**Fig. 7:**
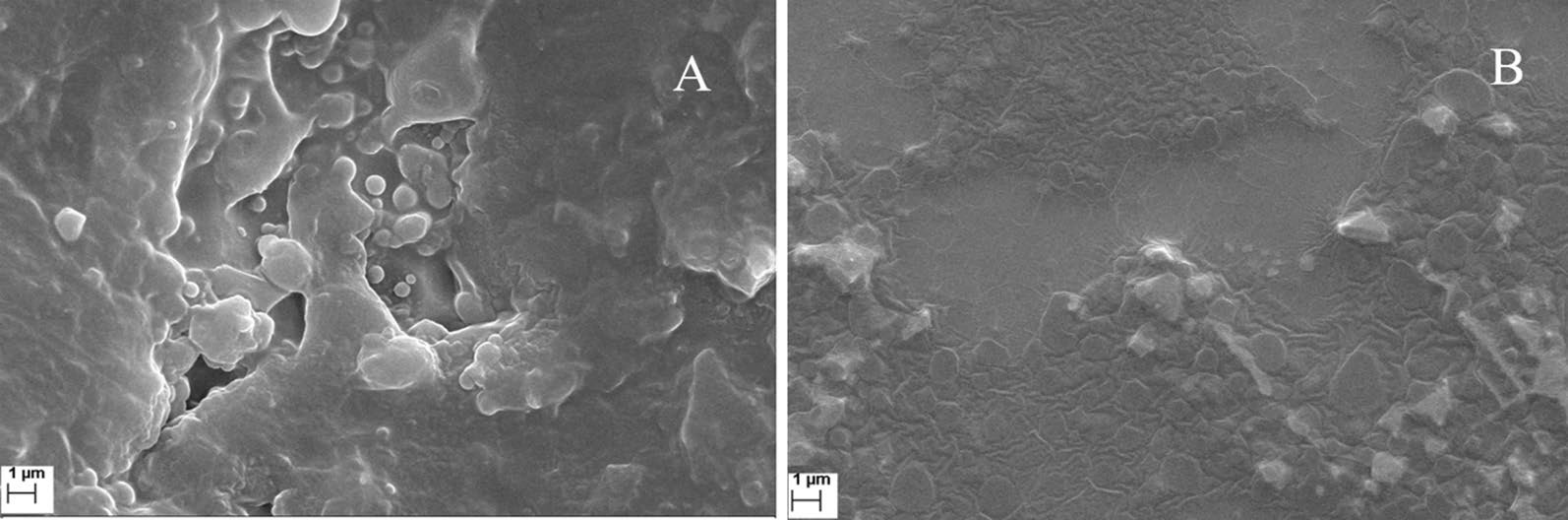
SEM images of TAE (**A**) and DAE (**B**) purified fraction

**Fig. 8:**
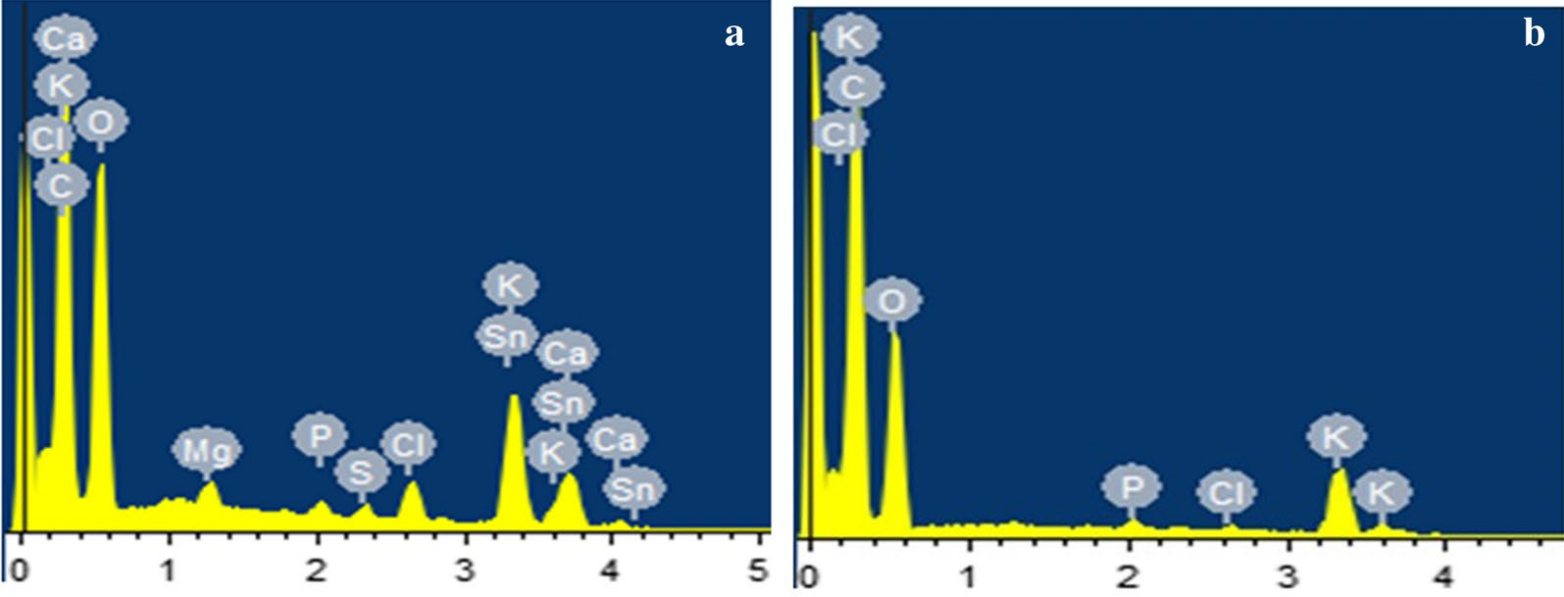
EDX of purified TAE (**a**) and DAE (**b**)

The morphology of the samples was observed under scanning electron microscope (SEM) with an accelerated voltage of 15 kV. SEM analyses showed that the purified samples were compact in nature with an uneven surface (Fig. 7). Also, it was not porous as observed in the samples extracted from TAE and DAE. The elemental analysis estimated that (Fig. 8) carbon, oxygen, magnesium, phosphorus, sulfur, chlorine, potassium, calcium, and tin are the elements mainly found in both extract (Table 3).

**Fig. 9:**
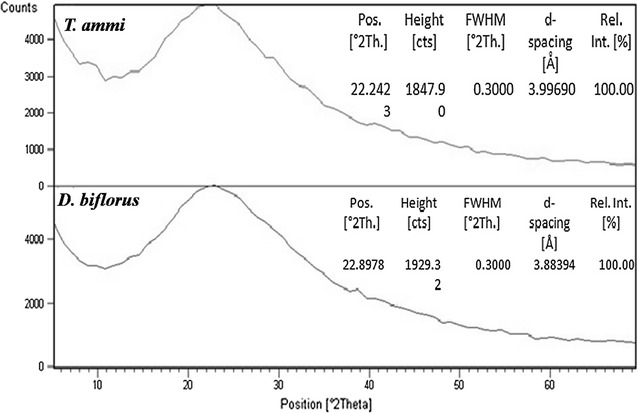
XRD profile of *T. ammi* (Linn) Sprague and *D. biflorus* Linn purified fraction of water extract

XRD analysis of samples represented the amorphous structure of the samples. As shown in Fig. 9, samples showed large hump distributed in a wide range (2θ) instead of high intensity narrower peaks with interplanner spacing (d-spacing) of 3.99 and 3.88 revealing the amorphous structure of the samples.

**Fig. 10:**
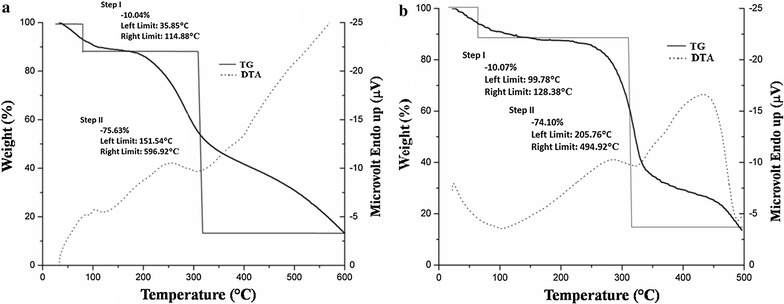
TGA and DTA response of (**a**) *T. ammi* and (**b**) *D. biflorus* purified extracts

Thermal gravimetric analysis or (TGA) measured changes in weight in relation to changes in temperature. TGA is commonly used to determine selected characteristics of materials that exhibit either mass loss or gain due to decomposition, oxidation, or loss of volatiles e.g. moisture. The TGA analysis showed degradation of TAE and DAE in two well-defined steps for both materials (Fig. 10). A total of 10.04 and 10.07% of weight loss for the first and 75.63 and 74.10% for the second step were recorded for samples TAE and DAE respectively. As discussed, weight loss during the first step below 100 °C indicated removal of moisture. From the results of DTA it can be clearly noticed that all the reactions were exothermic in nature.

**Fig. 11:**
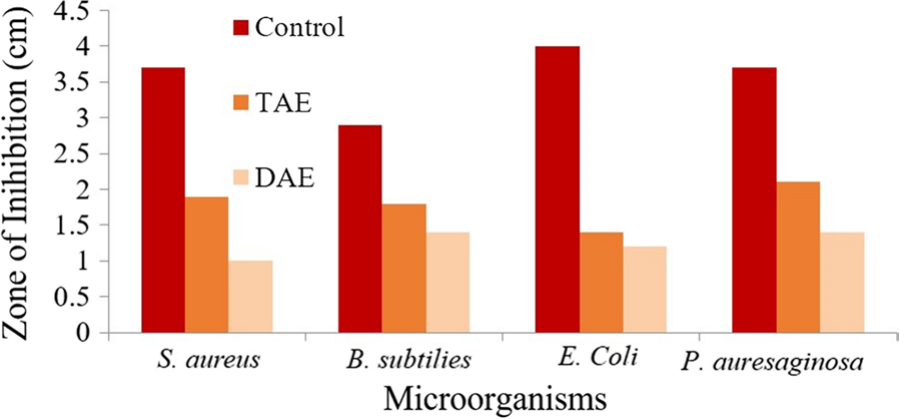
Antimicrobial activity against human pathogenic bacteria of TAE and DAE in comparison with positive control as tetracycline

**Fig. 12:**
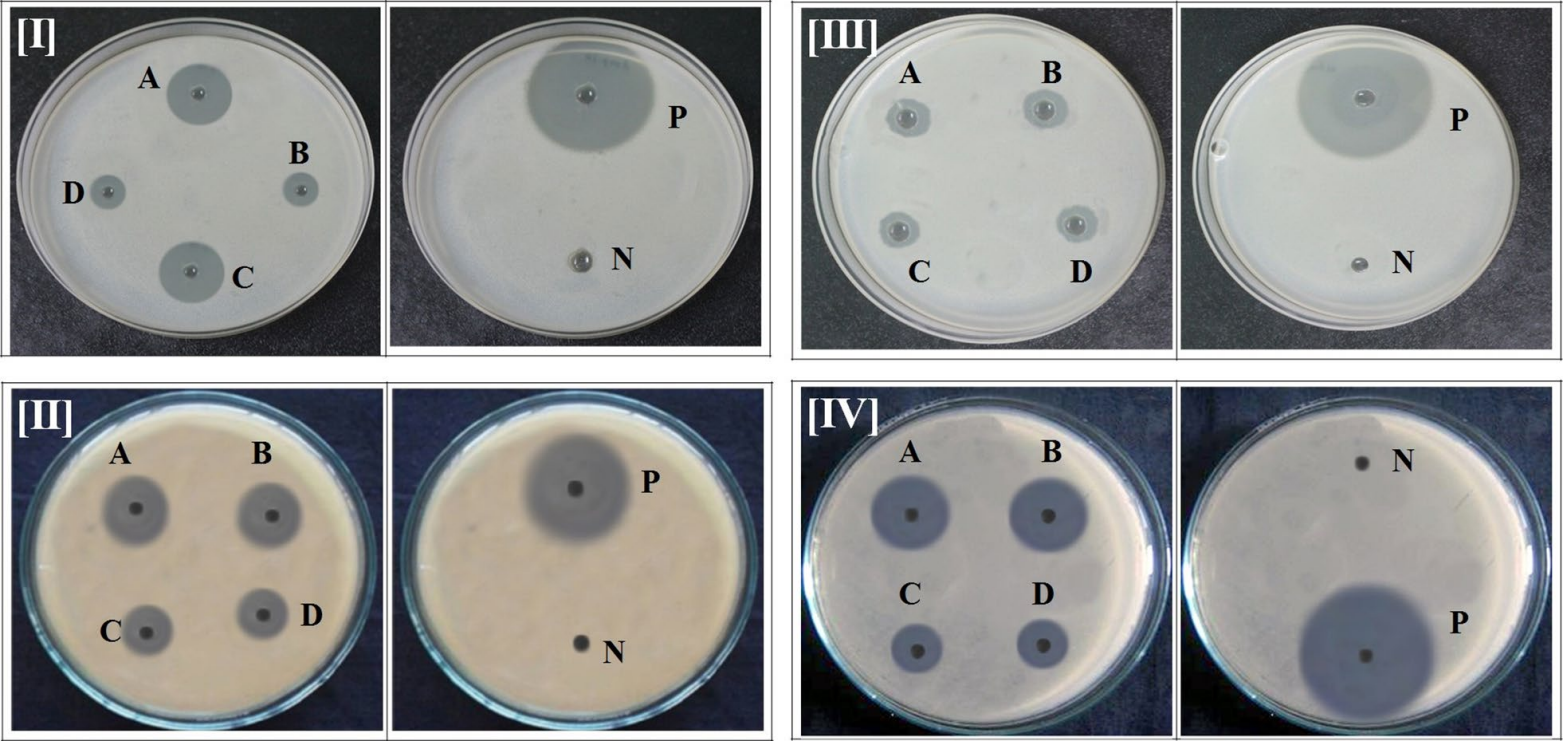
Antimicrobial activity against [**I**] *S. aureus* of TAE (A, C) and DAE (B, D); [**II**] *B.subtilis* of TAE (A, C) and DAE (B, D); [**III**] *E. coli* of TAE (A, C) DAE (B, D); [**IV**] *P. aureginosa* of TAE (A, C) DAE (B, D) in comparison with (P) positive control as tetracycline (N) negative control as normal saline water

Importantly it was detected that TAE and DAE have antibacterial activity (Fig. 11) against gram positive bacteria such as *Staphylococcus aureus, Bacillus subtilis*. In well diffusion method, TAE showed a maximum zone of inhibition against *Staphylococcus aureus* (1.9 cm) whereas DAE showed 1 cm zone of inhibition. A positive control (tetracycline) was used for comparative data and it showed 3.7 cm zone of inhibition. On the other hand, TAE showed zone of inhibition as 1.8 and 1.7 cm against *Bacillus subtilis* whereas, DAE showed 1.4 cm zone of inhibition. Extracts of both TAE and DAE showed significant antimicrobial activity with comparison to positive control (tetracycline) which showed 2.9 cm zone of inhibition. The zones of inhibition (cm) (Fig. 12) were reported in Table 4.

**Table 4:** Antimicrobial activity of purified extracts

Microorganism	Zone of inhibition (cm)		
	Control	TAE	DAE
*Staphylococcus aureus*	3.7 ± 0.12	1.9 ± 0.1	1 ± 0.08
		1.9 ± 0.14	1 ± 0.14
*Bacillus subtilis*	2.9 ± 0.11	1.8 ± 0.14	1.4 ± 0.15
		1.7 ± 0.11	1.4 ± 0.13
*Escherichia coli*	4 ± 0.14	1.4 ± 0.13	1.2 ± 0.13
		1.4 ± 0.12	1.2 ± 0.07
*Pseudomonas aeruginosa*	3.7 ± 0.14	2.1 ± 0.12	1.4 ± 0.14
		2.1 ± 0.09	1.4 ± 0.11

± Standard deviation

The above results were very encouraging and deserved further attention. It was gratifying to note that TAE and DAE also have antibacterial activity against Gram negative bacteria such as *Escherichia coli* and *Pseudomonas aeruginosa*. TAE showed a zone of inhibition as 1.4 cm and DAE showed 1.2 cm against *Escherichia coli*. Thus, the extracts of both *T. ammi* and *D. biflorus* showed antimicrobial activity in comparison to positive control (tetracycline) which showed 4 cm zone of inhibition. TAE showed the highest zone of inhibition against *Pseudomonas aeruginosa* (2.1 cm) among rest of microorganisms tested. DAE showed 1.4 cm zone of inhibition in comparison to positive control (tetracycline) which showed 3.5 cm zone inhibition. The molecular ratios of monosaccharide mixtures present in water soluble polysaccharide had significant effects on microbial activities. In this paper, TAE had shown better activity compared to DAE against gram positive bacteria (zone of inhibition in cm, Table 4). The percentage of aldehyde group (70%) seemed to have no effects on the antimicrobial properties of the extracts. The TAE extracts had more mannose than glucose. The DAE extracts had shown more or less equal activity against gram positive and gram negative bacteria. However, the DAE extracts had more glucose than mannose. In both of these extracts, it seemed galactose has no role. Glucose and mannose are epimeric. Ribose was present in the TAE extracts. However, no ribose was seen in the DAE extracts. In this study, we observe that extracts of TAE showed significant antimicrobial activity. The results of antimicrobial activity of these polysaccharides as demonstrated herein are new and novel. The effects polysaccharides against human sugar levels are studied by some authors. But those polysaccharides (TAE and DAE) can also act as promising antimicrobial agents are not explored.

## Conclusions

Compositions analysis for monosaccharide showed glucose, galactose and mannose residues are major compositions of the extract of TAE and a small amount of arabinose, ribose and xylose are also identified by GC–MS analysis. In DAE glucose and galactose are the main constituents. Also, the polysaccharides were morphologically nonporous as well as amorphous in nature. The antibacterial activities of TAE and DAE have a significant effect on human pathogenic bacteria. Among the both extracts, TAE showed better antimicrobial activity in comparison to the other extracts. So, extracts of TAE and DAE could be exploited for new potent antimicrobial agents.

## Abbreviations

GPC: gel permeation chromatoghrphy
SEM: scanning electron microscopy
XRD: X-ray diffractometer
EDX: energy-dispersive spectroscopy
FT-IR: the Fourier transform infrared spectra
TGA-DTA: thermo gravimetric and differential thermal analysis
GC-MS: gas chromatography mass spectrometry
TAE: *T. ammi* water extract
DAE: *D. biflorus* water extract
DCM: dichloromethane
